# Cucumber powdery mildew detection method based on hyperspectra-terahertz

**DOI:** 10.3389/fpls.2022.1035731

**Published:** 2022-09-29

**Authors:** Xiaodong Zhang, Pei Wang, Yafei Wang, Lian Hu, Xiwen Luo, Hanping Mao, Baoguo Shen

**Affiliations:** ^1^ College of Agricultural Engineering, Jiangsu University, Zhenjiang, China; ^2^ Key Laboratory of Modern Agricultural Equipment and Technology, Ministry of Education, Jiangsu University, Zhenjiang, China; ^3^ Key Laboratory of Key Technology on Agricultural Machine and Equipment, Ministry of Education, South China Agricultural University, Guangzhou, China

**Keywords:** cucumber, powdery mildew, hyperspectral, terahertz, disease detection

## Abstract

To explore the use of information technology in detecting crop diseases, a method based on hyperspectra-terahertz for detecting cucumber powdery mildew is proposed. Specifically, a method of effective hyperspectrum establishment, a method of spectral preprocessing, a method of selecting the feature wavelength, and a method of establishing discriminant models are studied. Firstly, the effective spectral information under visible light and near infrared is preprocessed by Savitzky-Golay (SG) smoothing, discrete wavelet transform, and move sliding window, which determine the optimal preprocessing method to be wavelet transform. Then stepwise discriminant analysis is used to select the feature wavelengths in the visible and near-infrared bands, forming the feature space. According to the features, a linear discriminant model is established for the wave bands, and the average recognition rate of cucumber powdery mildew is 93% in the whole wave band. The preprocessing method of terahertz data, the screening method of terahertz effective spectrum, the selection method of feature wavelength and the establishment method of classification model are studied. Python 3.8 is used to preprocess the terahertz raw data and establish the terahertz effective spectral data set for subsequent processing. Through iterative variable subset optimization - iterative retaining informative variables (IVSO-IRIV), the terahertz effective spectrum is screened twice to form the terahertz feature space. After that, the optimal regularization parameter and regularization solution methods are selected, and a sparse representation classification model is established. The accuracy of cucumber powdery mildew identification under the terahertz scale is 87.78%. The extraction and analysis methods of terahertz and hyperspectral feature images are studied, and more details of lesion samples are restored. Hence, the use of hyperspectral and terahertz technology can realize the detection of cucumber powdery mildew, which provides a basis for research on the hyperspectral and terahertz technology in detection of crop diseases.

## Introduction

China has the largest greenhouse area in the world at present. Cucumber is an important cash crop, and it is widely planted all over the world (Mao et al., 2022). Cucumber, as a functional food, has many nutritional values, high antioxidant capacity and high mineral content. In the process of cucumber cultivation in greenhouse, the high temperature and humidity environment in greenhouse can easily lead to diseases (Wang et al., 2022). Diseases will alter the physiological state of crops and affect their internal cells, pigment concentrations, moisture and intercellular gaps. Severe diseases also will threaten the ecoenvironment and food safety in addition to a decline of crop yields. Traditional plant disease detection methods rely on artificial sensory judgment, which calls for rich experience and observation ability of relevant personnel and is time-consuming. With the advancement of information technology, disease diagnosis and identification in target crops can be effectively conducted through the collection of disease case information from target crops.

The existing technologies such as machine vision, spectral detection and machine learning have been extensively studied in crop disease detection. Nilsson applied near-infrared spectroscopy for detection of rape rot and found the near-infrared reflectance was significantly correlated with rape rot severity (Nilsson, 1977). Maroua Nouri et al. detected hyperspectral images of healthy or infected apple tree leaves, and located the lesions of fruit tree leaves after reflection calibration and registration of hyperspectral images (Nouri et al., 2018). Tarek H M et al. proposed an online agricultural medical expert system based on image recognition and determined the disease after processing the images captured by mobile or hand-held devices, thereby helping farmers to solve problems (Habib et al., 2020). Chia-Lin Chung et al. put forward a method based on machine vision for nondestructive detection of disease infected or healthy seedlings, and classified healthy or infected seedlings by using a scanner and quantifying the shape and color features and through a support vector machine (SVM) algorithm, so as to realize disease detection (Chung et al., 2016). Du Xiuyang used a terahertz time-domain spectral technique and the least squares SVM (LS-SVM) to detect crop quality (Du et al., 2022). In all, spectral detection techniques featured by super bands and high resolution (Nilsson, 1977; Chung et al., 2016; Nouri et al., 2018; Habib et al., 2020; Wang et al., 2022) can accurately acquire inner spectral information of crops, and are especially outstanding in deteecting the absorption spectra of inner components of crops. The terahertz time-domain spectroscopy (Liu et al., 2020; Du et al., 2022) with penetrability can reflect crop changes at the terahertz scale from the transmission level.

This technique is widely applied into disease identification of field crops, but rarely used into detection of greenhouse plant diseases or detection at different scales, especially in disease recognition. Cucumber is a common widely-planted crop in the facility environment and highly caters to customers. In this study, cucumber was selectively investigated and a hyperspectral - terahertz method for detection of cucumber powdery mildew was explored.

## Materials and methods

### Sample culture

Cucumber was cultivated and sampled in the Venlo greenhouse of College of Agricultural Engineering, Jiangsu University in Zhenjiang of Jiangsu Province. Cucumber of “Jinyou 1” (provided by the cucumber research institute in Tianjin Academy of Agricultural Sciences, Tianjin, China) was bred soil-less from April to July 2019 in perlite. The average greenhouse air temperature was 21.6 ℃ (the range was from 11.32 to 37.73 ℃). The relative humidity of the greenhouse was 84.3%RH. The nutritive medium was a standard Hoagland composition. Upon the onset of cucumber powdery mildew, the front and back of leaves either showed nearly-round or continuous white powdery spots, and in severe cases, leaves will scorch and crisp. Totally 140 samples were acquired mainly through artificial collection, including 70 healthy samples and 70 powdery mildew samples.

### Data collection

**Figure 1:**
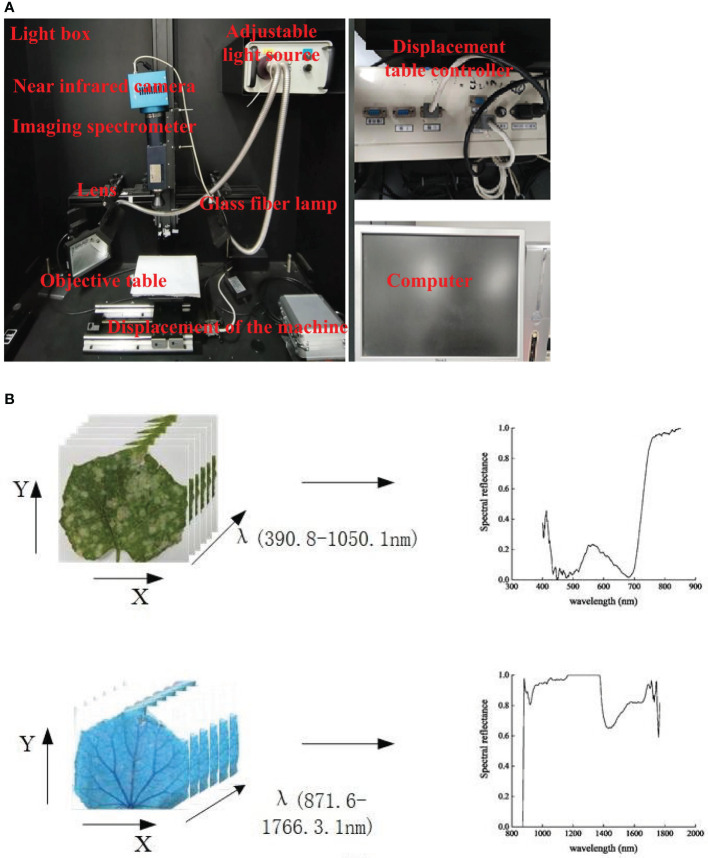
Hyperspectral imaging system. **(A)** Visible-near infrared hyperspectral equipment. **(B)** Visible-near infrared hyperspectral data acquisition.

A hyperspectral imaging system (Shanghai Wuling Photoelectric Technology Co., LTD) consisting of a visible light camera (VS, 390.8-1050.1 nm), a near-infrared camera (NIR, 871.6-1766.3 nm), an ImspectorN17E spectrometer, OLES30 lens, a direct- current adjustable light source, a glass fiber symmetrical line light source, a loading platform, a self-walking displacement platform, a stepping motor controller, a computer and a display was used here (**Figure 1**). Spectra of samples were recorded at the visible light band and the near-infrared band, and spectral data were stored in the three-dimensional form (x,y,λ) (**Figure 1B**). Presampling experiment was conducted prior to data acquisition. The hyperspectral imaging exposure time was 15 ms, the scanning rate was 1.32 mm/s, and the peak reflection intensity of leaf presampling images was 3000, which together ensured the clearness and non-distortion of images.

**Figure 2:**
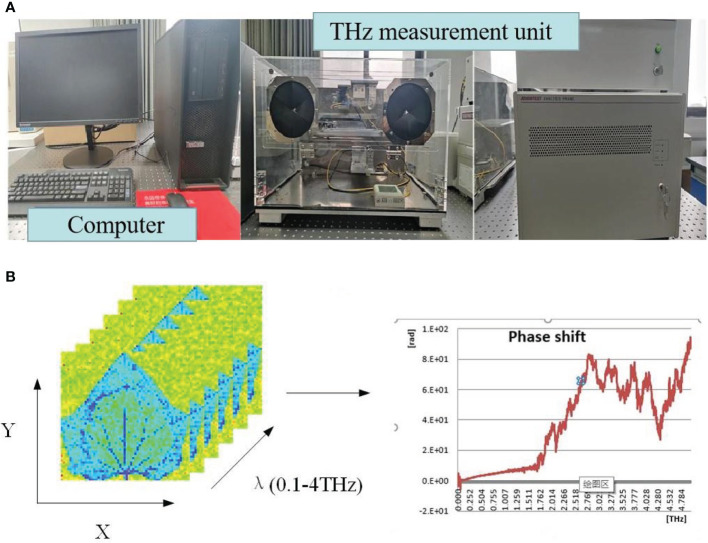
Terahertz time domain spectral system. **(A)** Terahertz experimental equipment. **(B)** Terahertz spectral data acquisition.

A TS7-400 Terahertz time-domain spectroscope (Advantest, Japan) was used here (**Figure 2A**). This instrument was customized and optimized in terms of crop bioinformatical detection, which improved the precision and enlarged the area of samples from 3 to 225 cm². Hence, this instrument satisfied the requirements for detecting most crop samples in the laboratory environment. The spectroscope can measure the absorption or transmission spectra at the frequency of 0-3.9 THz, and covered 1311 frequency ranges, with precision of 0.2 mm, signal-to-noise ratio of 5000 and spectral resolution of 3.8 GHz. The terahertz time-domain spectroscope consists of a terahertz measuring unit control system (keyboard, mouse, PC workstation) and a terahertz measuring unit (**Figure 2**).

Spectra data and terahertz data were acquired from the ill samples and healthy samples collected from the greenhouse by using the hyperspectral imaging system (including near-infrared and visible light bands) and the terahertz time-domain spectroscope. Disease-related data were acquired from several lesion areas of each ill leaf and specifically, the average spectral value and average terahertz value were determined from 5×5 uniformly- distributed pixels. For the healthy samples, each healthy leaf was divided into three parts (upper, middle and lower), and then several areas (each 5×5 pixels) from each part were detected to get the average spectral value. Totally 140 cucumber samples were collected, including 70 samples with powdery mildew and 70 healthy samples.

### Analysis of spectral data and modeling

The hyperspectral data of healthy or ill cucumber leaves were preprocessed by Savitzky-Golay (SG) smoothing, disperse wavelet transform, and move sliding window. The valid spectra screened out from the original spectra were compared to select the optimal preprocessing algorithm according to the preprocessed spectral curves and coefficient of determination (R^2^). The hyperspectral feature wavelengths were extracted and discriminated by using stepwise discriminant analysis (SDA). The feature images were processed analytically using gray extraction and pseudo-color rendering.

The terahertz data were first comprehensively extracted, and then a terahertz feature space was constructed by combining iteratively variable subset optimization (IVSO) and iteratively retaining informative variables (IRIV). A disease classification and identification model was built using a sparse representation classifier (SRC). The feature images were processed and analyzed by referring to the hyperspectral processing algorithm.

Data processing and classification modeling were accomplished on Matlab 2020a. Regression was conducted on SPSS. Feature image extraction and analysis were finished on the image processing software Python 3.8.

## Results and discussion

### Preprocessing of hyperspectral data and terahertz data

**Figure 3:**
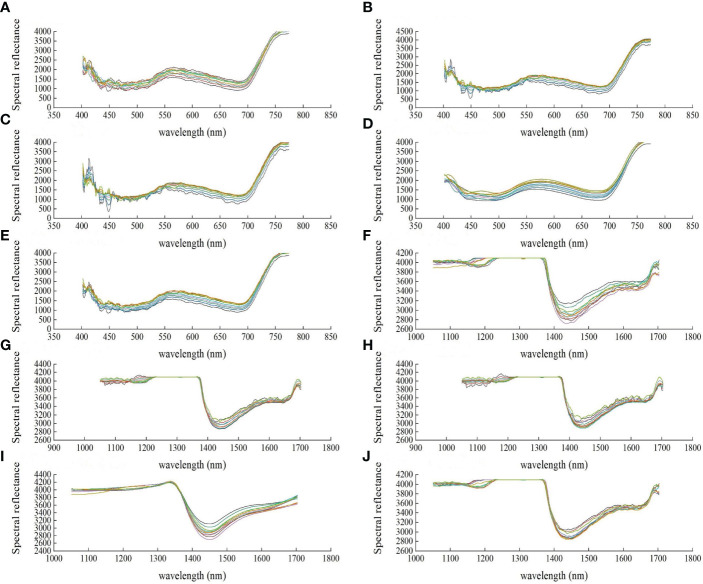
Spectral tlas of some cucumber disease amples under different preprocessing methods of hyperspectral data. **(A)** Visible original spectrum, **(B)** Visible spectrum SG7 point smoothing, **(C)**Visible spectrum SG9 point smoothing, **(D)** Visible spectrum discrete wavelet transform, **(E)** Visible spectrum moving window sliding, **(F)** Near infrared original spectrum, **(G)** Near infrared spectrum SG7 point smoothing, **(H)** Near infrared spectrum SG9 point smoothing, **(I)** Near infrared spectrum discrete wavelet transform, **(J)** Near infrared spectrum moving window sliding.

The spectral preprocessing algorithm can be selected according to the research target and spectrum type (Qin et al., 2020). Owing to spectral errors induced by the external environment or instruments, the collected data are often mixed with random noises, which decrease the data accuracy. Hence, spectra were preprocessed by SG smoothing, wavelet transform, or move sliding window. These three algorithms were compared by listing the processing results of a part of samples (**Figure 3**).

On the spectral curves processed by SG 7-point smoothing or 9-point smoothing, the strikes and shapes were similar to the original images, but some parts were distorted after noise processing, indicating noises were introduced there. Though moving-window can filter a part of noises, the processed curves are less disperse than the curves processed by wavelet transform and become partially overlapped, even with sharp tips in some of the data. As for disperse wavelet transform, the processed spectra are smooth, showing evident peaks and valleys, and the samples are not overlapped or concentrated and reserve the original features and rules. Together with the fitting in **Table 1**, the R^2^ of disperse wavelet transform is the highest (0.9964 and 0.96882). In all, disperse wavelet transform showed the best preprocessing effect and hence was adopted before feature extraction to preprocess the data acquired from cucumbers.

**Table 1:** Comparison of R-square (R^2^) after preprocessing.

Preprocessing	R^2^ (401.9-773.95nm)	R^2^ (1050.1-1703.40nm)
7-point SG smoothing	0.9871	0.9823
9-point SG smoothing	0.9747	0.9761
wavelet transform	0.9964	0.9882
move sliding window	0.993	0.9809

Different from hyperspectral data, the terahertz time- domain spectra were stored in the form of comma separated texts for each dot and background in single documents. Hence, the data of single folders was comprehensively extracted. The terahertz data were synthesized by Pandas in Python 3.8, which supports big data operation, into single documents for subsequent processing.

### Model building based on visible-near hyperspectral features

The key problem of hyperspectral imaging is to extract feature information from redundant spectral data and thereby to decrease the time and resource costs in subsequent processing. Hyperspectral data are located in a high-dimension space, and the data in each band can be considered as a feature. Hence, major subbands should be extracted from the spectral bands. In this study, n (n<491) subimage cubes were extracted from totally 491 valid bands covering visible light and near-infrared light and used as features.

**Figure 4:**
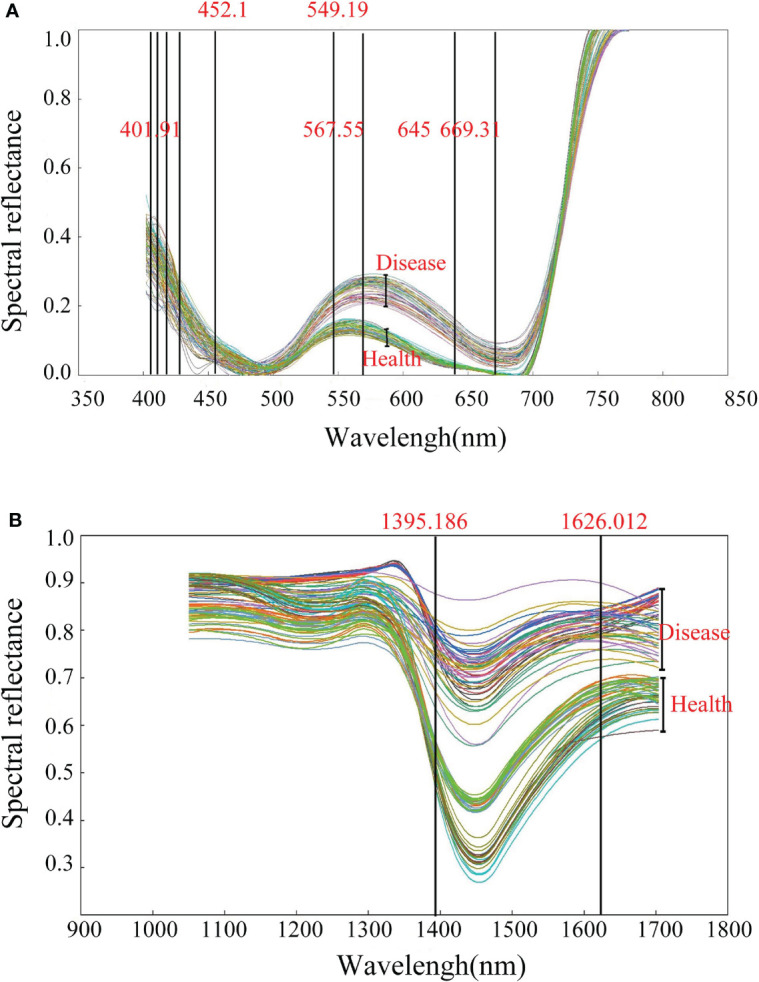
Feature wavelength distribution. **(A)** Visible light. **(B)** Near infrared.

SDA, a pattern recognition algorithm, is capable of extracting valid features from redundant spectral data (Chai et al., 2010). The cucumber leaf samples in the training set were processed by SDA in visible light (401.91 to 773.95 nm) and near-infrared light (1050 to 1703.40 nm). Finally, 9 visible light feature bands at 401.91, 403.14, 411.75, 429.01, 452.51, 549.19, 567.55, 645, and 669.31 nm were obtained, which were marked as R_401.91_, R_403.14_, R_411.75_, R_429.01_, R_452.51_, R_549.19_, R_567.55_, R_645_, and R_669.31_ respectively (**Figure 4A**). Two near-infrared feature bands at 1395.186 and 1626.012 nm were obtained, which were marked as R_1395.186_ and R_1626.012_ respectively (**Figure 4B**).

With the feature band spectral parameters, the powdery mildew samples and healthy samples were processed by distance discriminant analysis, which led to the discriminative models in **Tables 2** and **3** respectively. Based on these linear discriminative models, the samples in the training set were discriminatively tested. The correct discriminating rates of cucumber powdery mildew were 100% and 98% respectively **(** **Tables 4** and **5**). However, since the discriminative models were based on the training set, the identification effect may be exaggerated. For model validation, 40 extra datasets of healthy or powdery mildew cucumber samples were used as testing sets, and the correct identification rates in model validation were 95% and 93% respectively (**Tables 4** and **5**).

**Table 2:** Discriminant model of cucumber powdery mildew.

Type	Discriminant model (visible light)
Powdery mildew	Y1 = 128.824+956.526×R_401.91_-1045.165×R_403.14_+175.970×R_411.75_+159.199×R_429.01_+101.282×R_452.51_+77.351×R_549.19_+764.589×R_567.55_+322.834×R_645_-824.859×R_669.31_
Healthy	Y2=-104.507+1477.119×R_401.91_-1627.834×R_403.14_+305.193×R_411.75_+110.316×R_429.01_+465.589×R_452.51_+507.035×R_549.19_+198.567×R_567.55_-623.447×R_645_+37.900×R_669.31_

**Table 3:** Recognition model of cucumber powdery mildew in near infrared band.

Type	Discriminant model (near infrared)
Powdery mildew	Y1 = -283.955-0.153× R_1395.186_+0.301×R_1626.012_
Healthy	Y2 = -283.955-0.153× R_1395.186_+0.301×R_1626.012_

**Table 4:** Recognition results of cucumber powdery mildew (visible light).

Type	Sample number		Number of correct recognition		Rate of correct recognition/%	
	Training set	Test set	Training set	Test set	Training set	Test set
Powdery mildew	50	20	50	17	100	94
Healthy	50	20	50	18	100	96
Total	100	40	100	36	100	95

**Table 5:** Recognition results of cucumber powdery mildew (near infrared).

Type	Sample number		Number of correct recognition		Rate of correct recognition/%	
	Training set	Test set	Training set	Test set	Training set	Test set
Powdery mildew	50	20	48	16	98	92
Healthy	50	20	48	17	98	94
Total	100	40	100	36	98	93

### Model building based on terahertz spectral features

Like hyperspectra, the terahertz time-domain spectra also contain feature bands that are highly correlated with crop healthy and ill states. Herein, IVSO was used to reduce the dimensions of the whole valid terahertz spectra, which resulted in lower-dimension terahertz data that were highly correlated with the healthy and ill statuses of cucumber crops. These data were used into identification model establishment.

**Figure 5:**
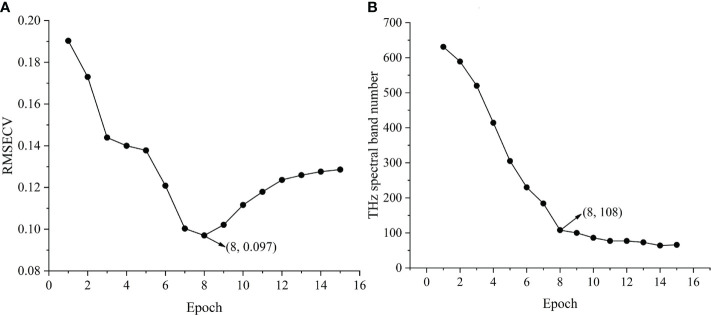
IVSO operation results. **(A)** RMSECV change curve in each iteration of cucumber sample. **(B)** THz band number change curve in each iteration of cucumber sample.

Before disease identification modeling, IVSO was used to reduce the dimensions of the whole valid terahertz spectra, which resulted in lower-dimension terahertz data that were highly correlated with the healthy and ill statuses of cucumber crops. These data were used into identification model establishment. From the valid terahertz spectral datasets, IVSO was used for the first time to screen out feature bands that were highly correlated the healthy and ill states of cucumber (Yun et al., 2014). IVSO was run on Matlab. Before running, relevant parameters of IVSO were set. The WBMS sampling times were set as the number of valid terahertz wave bands (631), the cross-validation pattern was default (10-fold), and the number of potential subset variables was 10. Results of IVSO were shown in **Figure 5**.

**Figures 5A, B** illustrates the root mean square error of cross-validation (RMSECV) of the partial least squares (PLS) model during the running of IVSO, and the curve of terahertz subband number changing with number of iterations, respectively. Clearly, RMSECV declined rapidly during the first 3 iterations, then minimized in the 8th iteration and gradually rose after that (**Figure 5A**). This was because IVSO during early iterations can remove abundant irrelevant variables and interfering variables. The decreased number of variables during late iterations mistakenly removed some useful variables, leading to the gradual rise of RMSECV during late iterations. The results of IVSO show the minimum RMSECV is 0.097, the optimal number of iterations is 8, and the number of feature bands is 108. The number of valid bands drops by 82.88% from the initial 631 valid bands, and at this moment, the number of feature dimensions is still large. Hence, IRIV was used to secondarily screen the terahertz feature bands. Relative to the single use of IRIV (Wang et al., 2015), the data at the terahertz feature bands screened out by IVSO, IRIV, or IVSO-IRIV were used as the training sets in PLS regression. The algorithm results of the feature bands as-selected were evaluated using the RMSECV and R^2^ of cross-validation. The number of feature bands in cucumber samples decreases to 28, RMSECV declines to 0.087, and R^2^ rises to 0.913 (**Table 6**), indicating modeling precision is improved and the number of irrelevant variables is decreased.

**Table 6:** Comparison of model results for terahertz feature wavelength selection.

Type	Algorithm	RMSECV		R^2^	Number of feature bands
Cucumber	IVSO	0.097		0.895	108
	IRIV	0.153		0.847	36
	IVSO-IRIV	0.096		0.913	28

**Figure 6:**
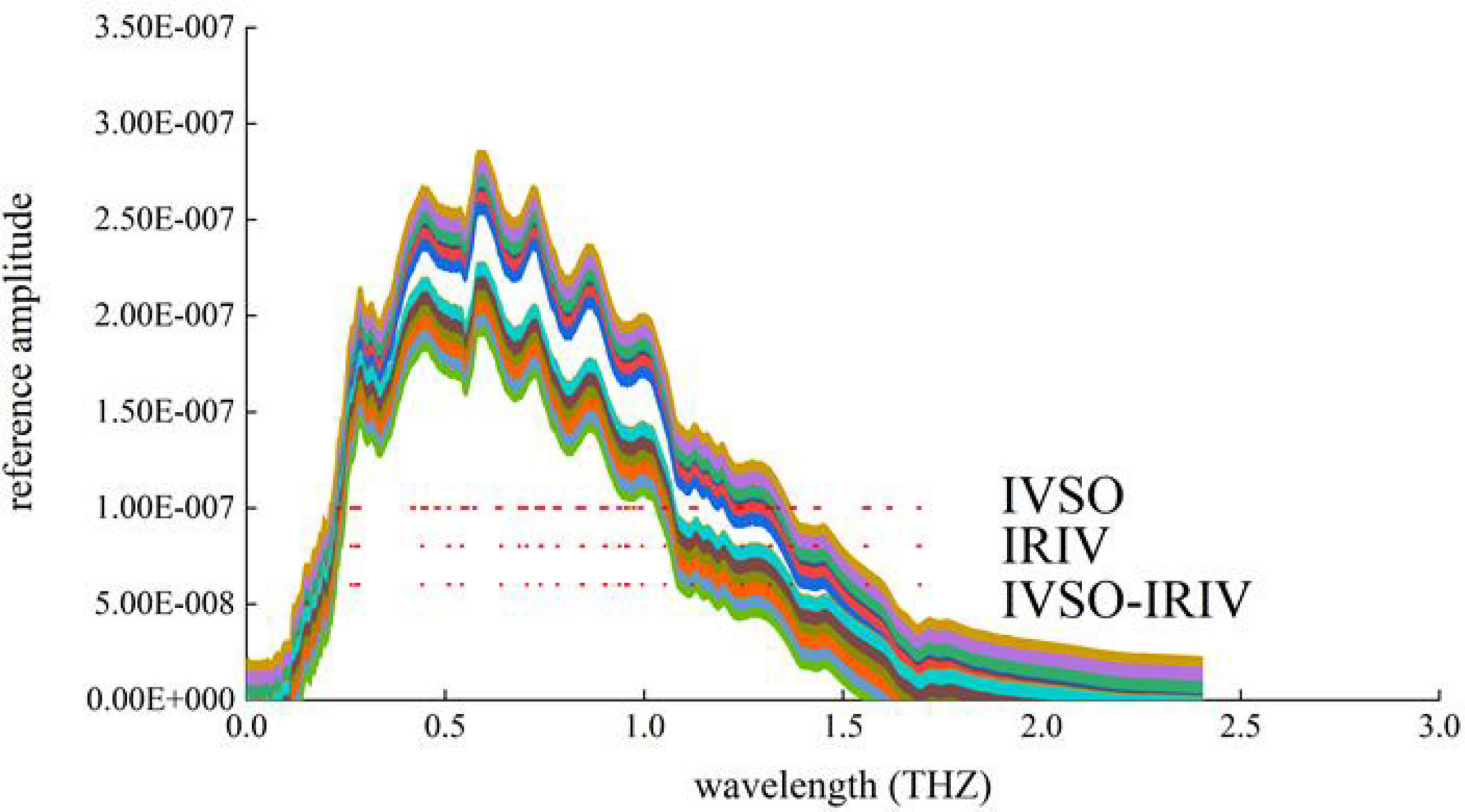
Terahertz characteristic wavelength distribution.

Based on the feature bands as-screened, the distributions of feature bands selected by IVSO, IRIV, and IVSO-IRIV were plotted (In **Figure 6**). Clearly, IVSO-IRIV effectively removed irrelevant variables and interfering variables. The terahertz bands selected by IVSO-IRIV are concentrated in 0.2-1.5 THz. After cucumber crops are infected by powdery mildew, the internal components (e.g. water, proteins, pigments) all will be altered to some extent. These components in this wave band are correlated with the penetrating ability of terahertz. Hence, from the feature bands selected by IVSO-IRIV, feature images were extracted, and a Terahertz-based disease identification model was built.

**Figure 7:**
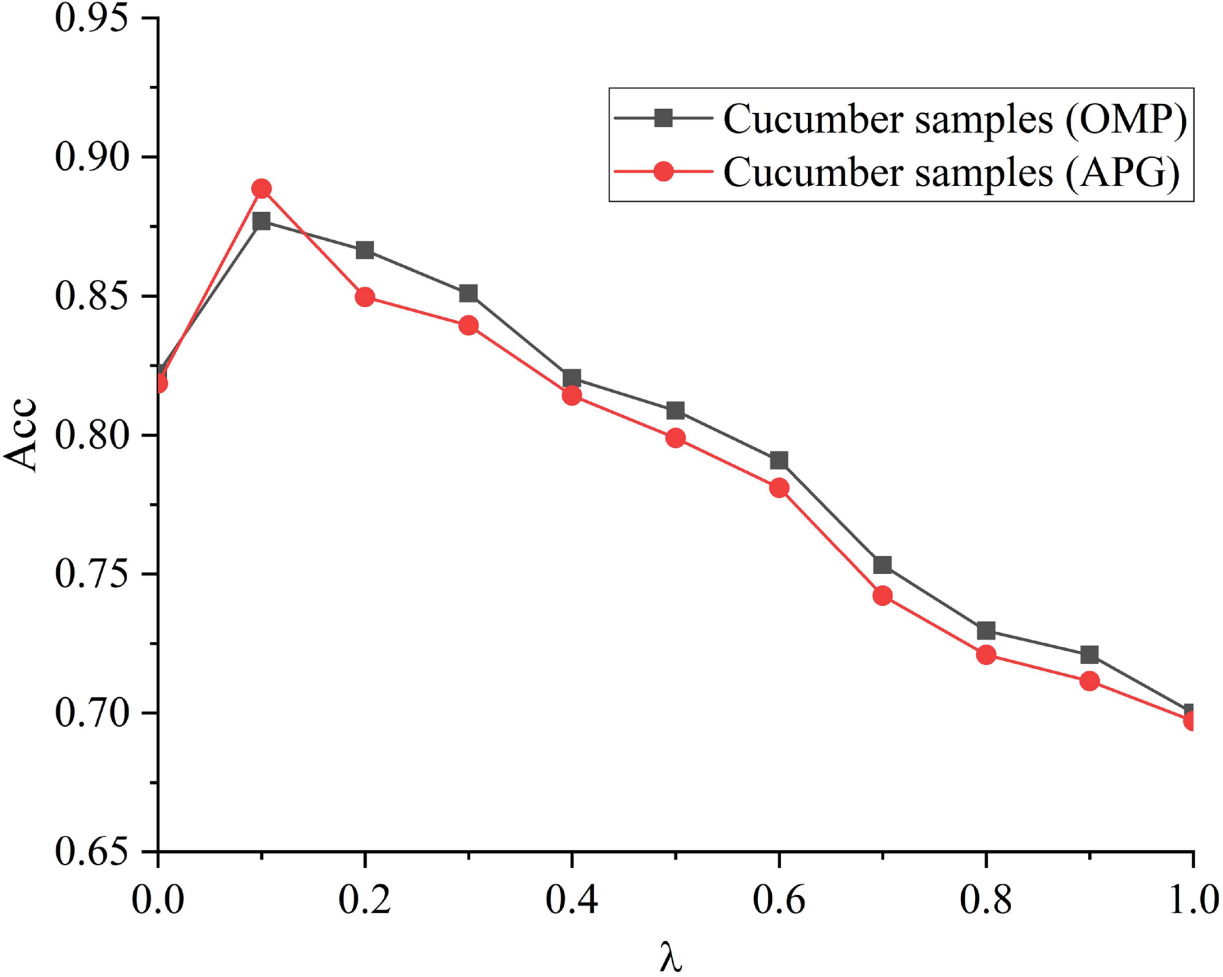
Influence of regularization parameters on recognition accuracy.

After the modeling, the processed data were inputted into the sparsity and dictionary learning toolbox SPAMS of Maltab (Wang and Cheng, 2020). A healthy model (positive samples) and an ill model (negative samples) were built, forming two training sets. Two redundant dictionary sets of the two training sets were constructed. Then each type of samples in the training sets were used to build redundant sub-dictionaries, and thereby discriminative dictionaries were built. Appropriate regularization parameter was chosen to optimize the redundant dictionaries. Namely, the effects of the regularization parameter on the models were evaluated using the parameters of 10-fold cross- validation. The optimal regularization parameter was determined to be 0.1. The sparse algorithms included orthogonal matching pursuit (OMP) and accelerated proximal gradient (APG). As λ increased, the result of 10-fold cross-validation decreased (**Figure 7**). This was because as the regularization parameter increased, the dictionaries were more sparse, and the number of relevant features declined, leading to the drop of classification accuracy. For different regularization algorithms, the identification accuracy of SRC models is 88.89% and 87.78%.

### Analysis of hyperspectral and terahertz feature images

**Figure 8:**
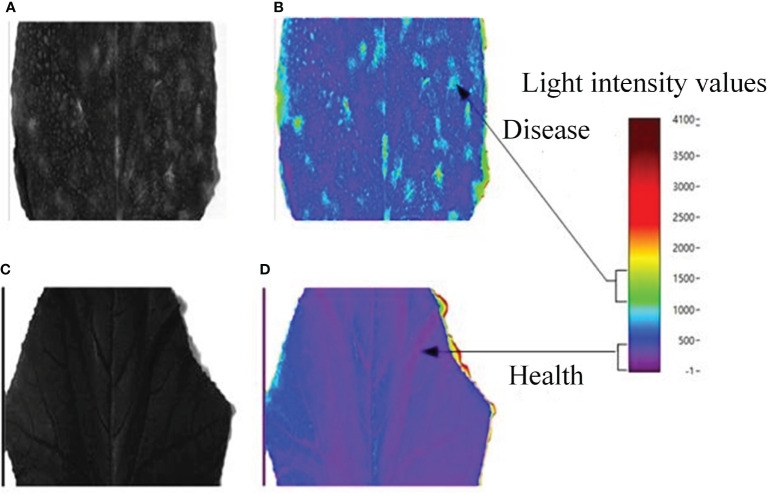
Characteristic images of visible light at 669.31nm: **(A)** Cucumber powdery mildew; pseudo-color images of **(B)** cucumber powdery mildew and **(D)** healthy cucumber samples; **(C)** Healthy samples of cucumbers.

Due to the large number of feature bands, we selected visible light band 669.31 nm and near-infrared band 1395.186 nm as two examples to process and analyze cucumber powdery mildew. **Figures 8A-C** shows the gray feature images at 669.31 nm of powdery mildew samples and healthy samples extracted from the hyperspectral images. Ill areas can be identified from the gray images, but the details are still insufficient. Thus, the gray images can be reconstructed into pseudo-color images, which can better identify more other details. Upon the infection by crop diseases, the surface and interior of crops are damaged to different degrees, and the optical reflection on crop surface also differs. To more precisely extract the lesion areas, we reconstituted the gray images of spectra into pseudo-color images by using a density stratification method. Into the hyperspectral extraction system built by Python, a transform function was added, and the intervals were appointed as light intensity distribution within 0-4096. With this method, pseudo-color images containing more details were obtained. Finally, pseudo-color images at visible light bands corresponding to 669.31 nm were obtained after image transformation (**Figures 8B-D**). Based on analysis of distributive scales of light intensity, the light intensity of lesion areas is distributed from 1000 to 1500, and that of healthy areas is distributed near 500. Moreover, some tiny differences that cannot be distinguished by human eyes on gray images are evident on the pseudo-color images, so that the features in lesion areas can be interpreted and extracted.

**Figure 9:**
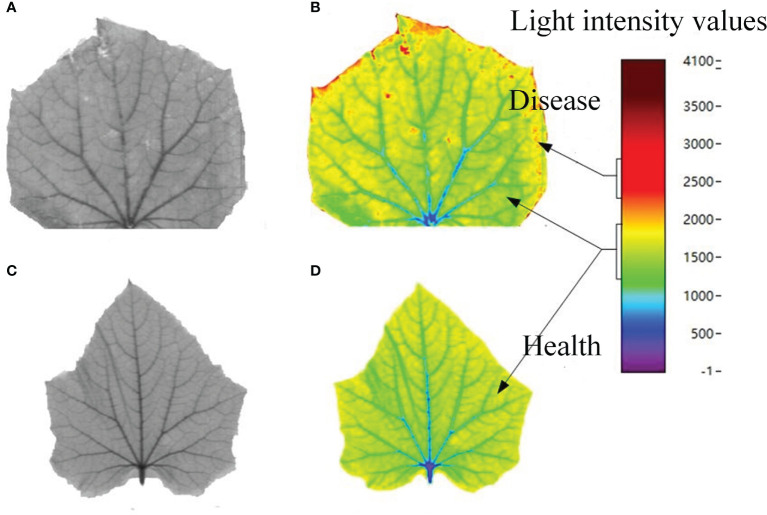
NIR characteristic images at 1395.186nm. **(A)** cucumber powdery mildew; pseudo-colour images of **(B)** cucumber powdery mildew and **(D)** healthy cucumber samples; **(C)** Healthy samples of cucumbers.

In **Figure 9**, with the same method, the features at near-infrared band 1395.186 nm were extracted and reproduced into pseudo-color images. Then distributions of healthy areas and lesion areas at the same light intensity scales were plotted, which returned more obvious features compared with the gray images.

**Figure 10:**
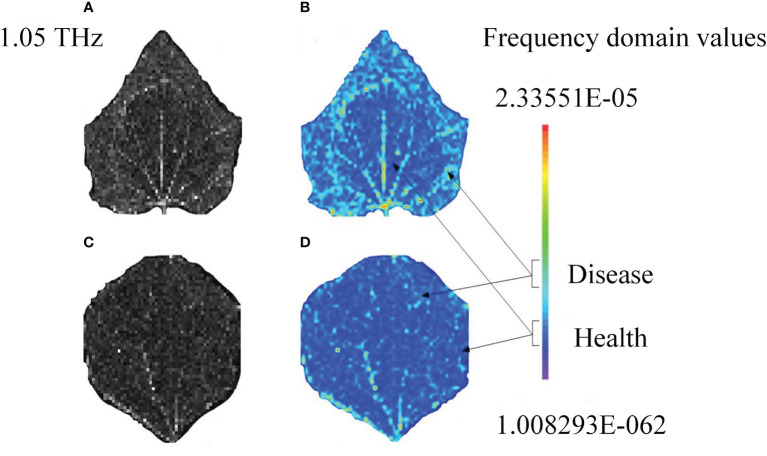
Terahertz characteristic images of cucumber powdery mildew: Feature grayscale images of **(A)** Sample 1 and **(C)** Sample 2; pseudo-colour rendering of **(B)** Sample 1 and **(D)** Sample 2.

Unlike the hyperspectral images, terahertz images have higher signal-to-noise ratio. Generally, frequency- domain amplitude imaging is adopted, and the signal value at a certain frequency selected from a frequency domain with large signal-to-noise ratio, and a frequency corresponding to the reference signal are chosen, and their ratio is regarded as a pixel value. In this way, the time-domain spectral data are converted by Fourier transform into frequency-domain data, which are used into imaging. Based on the designing clue of hyperspectral feature extraction, the terahertz feature images were extracted and plotted into images according to signal intensity. Finally, cucumber powdery mildew gray images at 1.05 THz were obtained (**Figures 10A-C**). The gray images demonstrate the outlines of ill samples, but cannot fully display the details. Therefore, given the advantages of false color images in displaying details, we obtained false color images by using frequency-domain intensity (**Figures 10B-D**). The gray images demonstrate the features of cucumber powdery mildew and tomato mosaic at the terahertz scale. However, the gray images are limited by low resolution and incomplete outlines and details. On the contrary, the false color images can recover the differences of crops in lesion areas and healthy areas according to the intensity of colors.

## Conclusions

The cucumber powdery mildew detection method based on hyperspectra and terahertz spectra underlies the disease detection in other facility crops. Starting from visible light and near-infrared bands, firstly wavelet transform was selected to be the optimal preprocessing algorithm. Then SDA was chosen to screen out feature wavelengths and to build detection models. As a result, the feature wavelengths under whole bands were obtained to constitute a feature space, and thereby linear discriminative models were established. The linear discriminative models were validated using the testing sets, and the recognition rate of cucumber powdery mildew was 93%. Based on terahertz time-domain spectroscopy, terahertz data were comprehensively preprocessed on Python, which improved the efficiency of subsequent data analysis. The terahertz feature space construction by IVSO-IRIV was determined, and SRC was tested. Thereby, an SRC model of cucumber powdery mildew was set up. Then the optimal regularization parameter was selected, and the effects of two regularization algorithms (OMP and APG) on model performances were compared. According to the evaluation criteria of disaggregated models, the identification accuracy of cucumber powdery mildew at the terahertz scale was 87.78%. Based on feature bands, a simple feature extraction system was established and used to uncover more details from the hyperspectral and terahertz feature images. In the future, the sample space should be enlarged, and more-precise identification models and multi-information fusion algorithms should be studied.

## Data availability statement

The original contributions presented in the study are included in the article/supplementary material. Further inquiries can be directed to the corresponding author.

## Author contributions

Conceptualization, XZ, LH, XL and HM; methodology, XZ, LH, XL and HM; formal analysis, PW and YW; data curation, XZ, PW, YW and BS; writing—original draft preparation, XZ and BS; writing—review and editing, XZ, LH, XL and HM; All authors contributed to the article and approved the submitted version.

## Funding

This research was funded by Project of Agricultural Equipment Department of Jiangsu University (NZXB20210106). Key Laboratory of Modern Agricultural Equipment and Technology(Jiangsu University), Ministry of Education (Grant No. MAET202111). National Key Research and Development Program for Young Scientists (2022YFD2000013). Key Laboratory of Modern Agricultural Equipment and Technology (Ministry of Education), High-tech Key Laboratory of Agricultural Equipment and Intelligence of Jiangsu Province (Grant No. JNZ201901). The National Natural Science Foundation of China (61771224 and 32071905).

## Conflict of interest

The authors declare that the research was conducted in the absence of any commercial or financial relationships that could be construed as a potential conflict of interest.

## Publisher’s note

All claims expressed in this article are solely those of the authors and do not necessarily represent those of their affiliated organizations, or those of the publisher, the editors and the reviewers. Any product that may be evaluated in this article, or claim that may be made by its manufacturer, is not guaranteed or endorsed by the publisher.
